# MiR-216a-5p inhibits tumorigenesis in Pancreatic Cancer by targeting TPT1/mTORC1 and is mediated by LINC01133

**DOI:** 10.7150/ijbs.46822

**Published:** 2020-07-19

**Authors:** Jian Zhang, Shuohui Gao, Yandong Zhang, Huixin Yi, Mengxian Xu, Jialun Xu, Huan Liu, Zhichen Ding, Hongbin He, Hongmei Wang, Zhuo Hao, Liankun Sun, Yan Liu, Feng Wei

**Affiliations:** 1 Department of Hepatobiliary and Pancreas Surgery, Jilin University First Hospital, Changchun, China.; 2 Genetic Engineering Laboratory of PLA, Institute of Military Veterinary Medicine, Academy of Military Medical Sciences, Changchun, China.; 3 Department of Gastrointestinal Colorectal Surgery, China-Japan Union hospital of Jilin University, Changchun, China.; 4 Department of Pathophysiology, College of Basic Medicine Sciences, Jilin University, Changchun, China.; 5 Key Laboratory of Zoonosis Research, Ministry of Education, College of Veterinary Medicine, Jilin University.; 6 Ruminant Diseases Research Center, College of Life Sciences, Shandong Normal University, Jinan, China.

**Keywords:** LINC01133, pancreatic cancer, miR-216a-5p, TPT1, tumor progression

## Abstract

MiR-216a-5p has opposite effects on tumorigenesis and progression in the context of different tumors, acting as either a tumor suppressor or an oncogene. However, the expression and function of miR-216a-5p in pancreatic cancer (PC) is not well characterized.

In this study, we found miR-216a-5p was significantly downregulated in PC tissues and cell lines, which showed a negative correlation with peripancreatic lymph, perineural invasion and TNM stage of PCs patients. We made use of functional assays to reveal that miR-216a-5p inhibited growth and migration of PC cells *in vitro* and *in vivo*. Then, by employing the bioinformatics analysis and luciferase reporter assay, we demonstrated TPT1 was a potential target of miR-216a-5p, which contributes to tumor malignance by mediating mTORC1 pathway-associated autophagy. Furthermore, bioinformatics analysis and RNA pulldown confirmed that miR-216a-5p was mediated by LINC01133, which sponge miR-216a-5p, as a competing endogenous RNA (ceRNA). Collectively, our study revealed an important role of LINC01133/miR-216a-5p/TPT1 axis in the genesis and progression of PCs, which provides potential biomarkers for clinical diagnosis and therapy of PCs.

## Introduction

Pancreatic cancer (PC) is one of the most fatal solid tumors with over 57,600 new cases and approximately the same number of deaths reported every year globally. It is currently the fourth cancer-related deaths in the United States and the eighth male cancer-related morbidity in China 1, 2. In past decades, there has been a steady increase in the survival rates for most cancer types. However, the 5-year survival rates of PC patients have remained at the lowest of 9%. Despite the identification and characterization of numerous tumor biomarkers and molecular mechanisms, the dismal prognosis is largely attributable to delayed diagnosis and drug resistance.

Non-coding RNAs including both microRNAs (miRNAs) and long non-coding RNAs (lncRNAs) are aberrantly expressed in different types of cancers. These miRNAs play a key role in the initiation and progression of tumors. Indeed, the role of miRNAs as clinical biomarkers and therapeutic targets has been widely investigated. MiRNAs usually bind with the 3'-UTR of target genes and repress gene expression during the post-transcriptional process by inhibiting translation or promoting mRNA degradation. LncRNAs are conserved RNA transcripts with more than 200nt that lack protein-coding potential, which were reported to drive important cancer phenotypes by interacting with proteins, RNAs, and DNAs. In addition, LncRNAs can also interact with their specific miRNAs like sponges and inhibit the expression of target genes in a miRNA-dependent manner. For example, lncRNA *MEG3* was shown to enhance the transcriptional activation of p53 and to downregulate* MDM2*, resulting in cell-cycle arrest and apoptosis as well as modulation of autophagy 3. Further, lncRNA *ELIT-1* acts as a Smad3 cofactor to facilitate TGF-β/Smad signaling and promotes epithelial-mesenchymal transition (EMT) 4. LncRNA *CAR10* promotes lung adenocarcinoma metastasis* via* the miR-203/30/SNAI axis 5. LncRNA *SNHG5* was shown to promote progression of hepatocellular carcinoma by regulating miR-26a-50/GSK3β signaling pathway 6. MiR-216a-5p was shown to act as a tumor suppressor in breast cancer by targeting PAK2 and be regulated by lncRNA DANCR 7. Conversely, miR-216a-5p acts as an oncogene in renal cell carcinoma 8 and hepatocellular carcinoma, and was shown to be regulated by lncRNA-A1BG-AS1, as a ceRNA 9. Although miR-216 cluster was found low expressed in pancreatic ductal adenocarcinoma by RNA sequencing 10, the role of miR-216a-5p in the initiation and progression of PC is not well characterized.

TPT1/TCTP (tumor protein, translationally-controlled 1) is a conserved onco-protein that is overexpressed in several human cancers, and participates in multiple cellular activities including protein synthesis, cell survival and malignant transformation. TPT1 could work as a functional target of P53 that promotes cell survival 11-13; TPT1 was shown to negatively regulate autophagy through the BECN1 interaction and mTORC1-mediated pathway 14. In addition, TPT1 may also be regulated by miR-145-5p and miR-27b in nonfunctioning pituitary adenomas and oral cancer, respectively 15, 16. The present study explored whether TPT1 is a target of miR-216a-5p and whether it is involved in miR-216a-5p mediated onco-functions.

In the current study, we demonstrated that miR-216a-5p is strongly downregulated in PC tissues and cells, and functions as a tumor suppressor. Its downregulation was closely associated with poor prognosis and metastasis in PC patients. Overexpression of miR-216a-5p potentially prohibited cell proliferation and metastasis, and decreased tumorigenicity and metastasis in SCID mice. Moreover, miR-216a-5p exerted its anti-tumor functions by specifically inhibiting TPT1 and was found to be associated with the mTORC1 signaling pathway. Importantly, miR-216a-5p was negatively regulated by LINC01133. Collectively, our study identified that the LINC01133-miR-216a-5p-TPT1 axis effectively contributed to the tumorigenesis and progression of PC. Thus, our findings may provide new strategies for the diagnosis and treatment of PC.

## Methods

### Patients and tissue samples

Forty pairs of PC tissues and adjacent normal tissues (ANTs) were surgically resected from patients at the First Affiliated Hospital of Jilin University. The central parts of these tissues, about 4mm in diameter, were immediately stored in liquid nitrogen until further assays. None of these patients had received chemotherapy or radiotherapy prior to surgery. Table 1 shows the clinical and pathological characteristics of patients. This study abided by the Declaration of Helsinki was approved by the Institutional Ethics Committee of the First Affiliated Hospital of Jilin University. Written informed consent was obtained from all patients before the research.

### Cell culture and transfection

PC cell lines (SW1990, PANC1, Capan-2 and BxPC-3) and human pancreatic ductal epithelial cell line (HPDE6) were purchased from Genechem (Shanghai, China). The cell lines have been tested for mycoplasma contamination. All cells were cultured in a humidified incubator at 37 °C with 5% CO_2_. The following complete growth media were used as per the recommendations: Dulbecco's Modified Eagle Medium/F12 (DMEM/F12), Dulbecco's Modified Eagle Medium (DMEM), and Roswell Park Memorial Institute (RPMI) 1640 (Corning, Manassas, USA), supplemented with 10% fetal bovine serum (FBS) (BI, USA) and 1% penicillin and streptomycin (Gibco, Grand Island, NY). All human cell lines have been authenticated using STR profiling within the last three years and all experiments were performed with mycoplasma-free cells. With the help of Lipofectamine 2000 (Invitrogen, CA, USA), LINC01133 siRNA, LINC01133 over-expressing plasmid, miR-216a-5p mimic/inhibitors, TPT1 siRNA (Genepharma, Shanghai), pcDNA3.1(+)-TPT1 over-expressing plasmid and the corresponding negative controls were transfected into different cells according to the manufacturer's protocol. The transfected cells were collected for further assay after 48 hours. Stable SW1990-216^KD^-*luc*2, SW1990-miR^KD^-ctrl-*luc*2, SW1990-216-*luc*2, and SW1990-miR-ctrl-*luc*2 cell lines were established using lentivirus Ubi-LUC-MCS-IRES-puromycin with negative control or oligonucleotides against miR-216a-5p (GeneChem). The cells were continuously incubated with puromycin (1.0 μg/mL, Sigma) to allow the development of acquired resistance.

### RNA extraction and qRT-PCR analysis

TRIzol reagent (TAKARA, China) was used to extract total RNA from cells and tissues. cDNA was synthesized from 20 μg RNA using Reverse Transcription Kit with either Random primer or Bulge-Loop^TM^ miRNA RT-PCR primers (RIBOBio, Guangzhou). Quantitative PCR was performed using SYBR qPCR Master Mix to assess the expression levels of miR-216a-5p, LINC01133 and TPT1. U6 and GAPDH were used as internal controls for data normalization. All PCR experiments were performed in triplicate. The sequences of primers are as follows:

- LINC01133, Forward primers: 5'-ATGGTTGGGAGGAGGTAAA-3',
- Reverse primers: 5'-TGGGCTCAAGGAATCTGAATA-3';
- TPT1, Forward primers: 5'-CTTTCTGTCTGTCTTTGTGGCG-3',
- Reverse primers: 5'-AGTCCTGGTGTTGTGTGGATG-3';
- GAPDH, Forward primers: 5'-GACAGTCAGCCGCATCTTCT-3',
- Reverse primers: 5'-GCGCCCAATACGACCAAATC-3'.

### Western blot analysis

Total proteins were extracted using lysis buffer (RIPA, Millipore) with 1% protease inhibitor cocktail (Thermo Scientific, USA). Equal amounts of protein samples were separated by SDS-PAGE and transferred to PVDF membrane. Membranes were blocked by Odyssey blocking buffer, then incubated overnight with primary antibodies including those against TCTP/TPT1 (Sino Biological, 14662-T60), mTOR (p/T) (Cell Signaling Technology, CST, 5536S/2983S), LC3 (Sigma-Aldrich, ABC929), ULK1(p/T) (CST, 14202T/4776S), P70S6K(CST, 9204S) and p62 (CST, 8025). After incubation with HRP-conjugated secondary antibodies, the membranes were washed and the bands were visualized using the Tanon system (Tanon, China); α-tubulin (Beyotime, AF0001) was used as an endogenous reference.

### EdU incorporation assay for cell proliferation

EdU cell proliferation kit (RIBOBio, Guangzhou) was used to measure cell proliferation. In brief, cells were seeded into 12-well plates and transfected as previously described. After 36 hours, cells were incubated in serum-free medium supplemented with EdU (50 μM) for 2 hours; subsequently, washed thrice in PBS. This was followed by fixation with 4% paraformaldehyde for 30 min at room temperature. After neutralization with extra paraformaldehyde with glycine, cells were stained with Apollo mixture and Hoechst 33342 for 30 min in dark. Finally, cells were washed with PBS and images were captured using microscope (Olympus, Japan). The proliferation index was calculated as the percentage of EdU-positive cells relative to the total cell numbers in at least five random fields.

### Cell viability assay

The sulforhodamine B (SRB) assay was performed to determine cell viability. Briefly, cells were seeded into 96-well plates at 8×10³ /well and cultured overnight. Cells were treated with different anti-tumor drugs (gemcitabine, doxorubicin) for 48 hours at 37 °C and fixed with 10% cold trichloroacetic acid (TCA) for 45 min, followed by staining with 0.4% SRB for 15 min. After washed with acetic acid, the dye was resolved by 10 mM Tris-base buffer. The absorbance was measured with a spectrophotometer at 510 nm and cell viability was calculated using the equation: % cell viability = (At/Ac) ×100%, where At and Ac represent the absorbance in treatment and control cultures, respectively.

### Colony formation assay

1×10^3^ indicated cells were planted in 6-well plates and the medium was replaced every 3 days. 12 days later, when colonies were formed obviously, cells were fixed by 4% paraformaldehyde for 30 min. Subsequently, colonies were stained with crystal violet. Cell colonies were photographed and counted.

### Wound healing assay

Wound healing assay was carried out to evaluate the cell migration ability. Cells were treated as indicated and seeded into 6-well plates. After achievement of 80% confluence following overnight culture, a scratch was made with sterile pipette tip and the cells were incubated in complete medium with 2% FBS (BI, USA) to minimize proliferation. Images were obtained by microscope (Olympus, Japan) 12 hours after inflicting the wound.

### Transwell assay

Cell invasion assay was carried out using Transwell chambers with 8 μm pores (Costar, USA). In brief, transfected cells (1×10^5^) in serum-free medium were seeded in the upper insert and 600 μL complete medium with 10% FBS was added in the bottom chamber as chemoattractant. After 24 hours of incubation, the upper surface of each membrane was carefully cleaned, and the cells that were adhered to the insert surface were fixed with 4% paraformaldehyde and stained with DAPI.

### Flow cytometry assay

For cell cycle analysis, transfected cells were cultured for 24 h and cell suspension was prepared. After washed with cold PBS, the collected cells were fixed with 70% cold ethanol at 4℃ overnight, then stained with PI mixture (BD Biosciences, San Diego, USA) according to the manufacturer's instructions. Data were acquired using flow cytometry and analyzed with FlowJo software.

### Luciferase reporter assay

The dual-luciferase vectors were constructed by synthesizing the seed sequences of the 3'-UTR of TPT1 or the reverse complementary sequence of miR-216a and inserting these into psiCHECK-2 vectors. The corresponding mutant vectors were constructed by introducing 3-bp mutations into the above seed sequences. To verify the specific targeting of TPT1 by miR-216a-5p, HEK-293 T cells were seeded in 24-well plates and transfected with 0.8 μg of recombinant vectors, either alone or with miR-216a-5p mimic or inhibitor. Firefly and Renilla luciferase activities in cell lysates was measured 24 h later using the dual-Glo reporter assay system (Promega, Madison, USA).

### RNA pull down assay

The DNA fragments of the full length LINC01133 was PCR amplified using T7-containing primer and transcribed *in vitro* by using the T7 High Yield RNA Synthesis Kit's (Thermo, Waltham, MA). Obtained LINC01133 RNAs and standard RNA were labeled using 3' End Biotinylation Kit (Thermo, Waltham, MA). Subsequently, Biotin-labled RNA pull-down assay was carried out to detect the interact miRNAs (Thermo, Waltham, MA). Briefly, labled-lncRNAs were captured to streptavidin magnetic beads in Capture Buffer for 30 min at room temperature with agitation. Meanwhile, indicated cell lysates were collected, and mixed with lncRNA-conjugated beads for 1 h at 4 °C on a rotator. After the beads were washed thoroughly, the beads-bound RNA was isolated and subjected to qRT-PCR analysis. Input RNA was extracted and served as control.

### Fluorescence in situ hybridization (FISH)

FISH assay was performed in HPDE6, BxPC-3 and SW1990 cell lines to detect the distribution of LINC01133. Cy3-labled LINC01133 probes were designed and synthesized by Ribio (Guangzhou, China). Briefly, the indicated cells were fixed with 4% paraformaldehyde for 10 min at room temperature. After permeabilization, cells were hybridized with probes overnight at 37 °C. After cleansing, nuclei were stained with DAPI for 10 min. The images were captured by Olympus microscope.

### Nuclear/cytoplasmic fractionation assay

The separation of nuclear RNA from the cytoplasmic fraction was achieved by following the instructions of nuclear/cytosol fractionation kit. Briefly, collected cells were mixed with cytosol extraction buffer vigorously. After a 5-minute centrifugation, supernatants (cytoplasmic extract) were collected. The pellets were then resupended with nuclear extraction buffer mix. After vortex for 15 seconds every 10 minutes for a total 40 minutes, nuclear extracts were collected. Total RNA were extracted from these tubes and were examined in further assays.

### *In vivo* lung metastasis model

Animal experiments were performed according to the protocol approved by the Institutional Animal Care and Use Committee of Jilin University. First, 1×10^6^ cells of the stable cell lines SW1990-216^KD^-*luc*2, SW1990-miR^KD^-ctrl-*luc*2, SW1990-216-*luc*2, and SW1990-miR-ctrl-*luc*2 cells were injected into randomly-divided four groups of severe combined immunodeficient (SCID) mice *via* the tail vein (n=32, female) (Vital River, Beijing), followed by instant luciferase imaging to confirm that the injection was successful. To achieve this, mice were anesthetized and injected intraperitoneally with luciferin (25 mg/mL in 0.1 mL PBS), and images were collected beyond 15 min post-injection using the IVIS LuminaXR system (Caliper, Hopkinton, MA, USA). Lucifer emission from the tumor tissues were assessed and light emission (photons/s) was measured using software provided by the vendor (Xenogen, Corp, Alameda, CA, USA). The images were obtained once a week for a total of 6 weeks. Finally, all mice were sacrificed and tumor metastasis tissues were obtained for further experiments.

### Immunohistochemistry

The paraffin sections of tissues resected from enrolled PC patients were dewaxed and rehydrated. Then, citrate buffer and 3% H_2_O_2_ were respectively used to retrieve antigen and block the endogenous peroxidase activity. After blocking with 0.5% BSA, the samples were incubated with TCTP/TPT1 antibody (1:50) at 4 °C overnight, and secondary antibody (MaxVision HRP-polymer anti-Rabbit IHC Kit) for 30 min at room temperature. The sections were stained with DAB and hematoxylin. Images were captured using Olympus microscope.

### Statistical analysis

All statistical analyses were conducted using GraphPad Prism software (GraphPad, Inc, San Diego, CA). Data are presented as mean ± standard deviation (SD). Two-tailed unpaired Student's *t*-test was used to assess differences between two groups. The clinicopathological parameters of patients were analyzed by Fisher's exact test. *P* values < 0.05 were considered indicative of statistical significance.

## Results

### MiR-216a-5p is downregulated in PC tissues and cell lines

**Figure 1:**
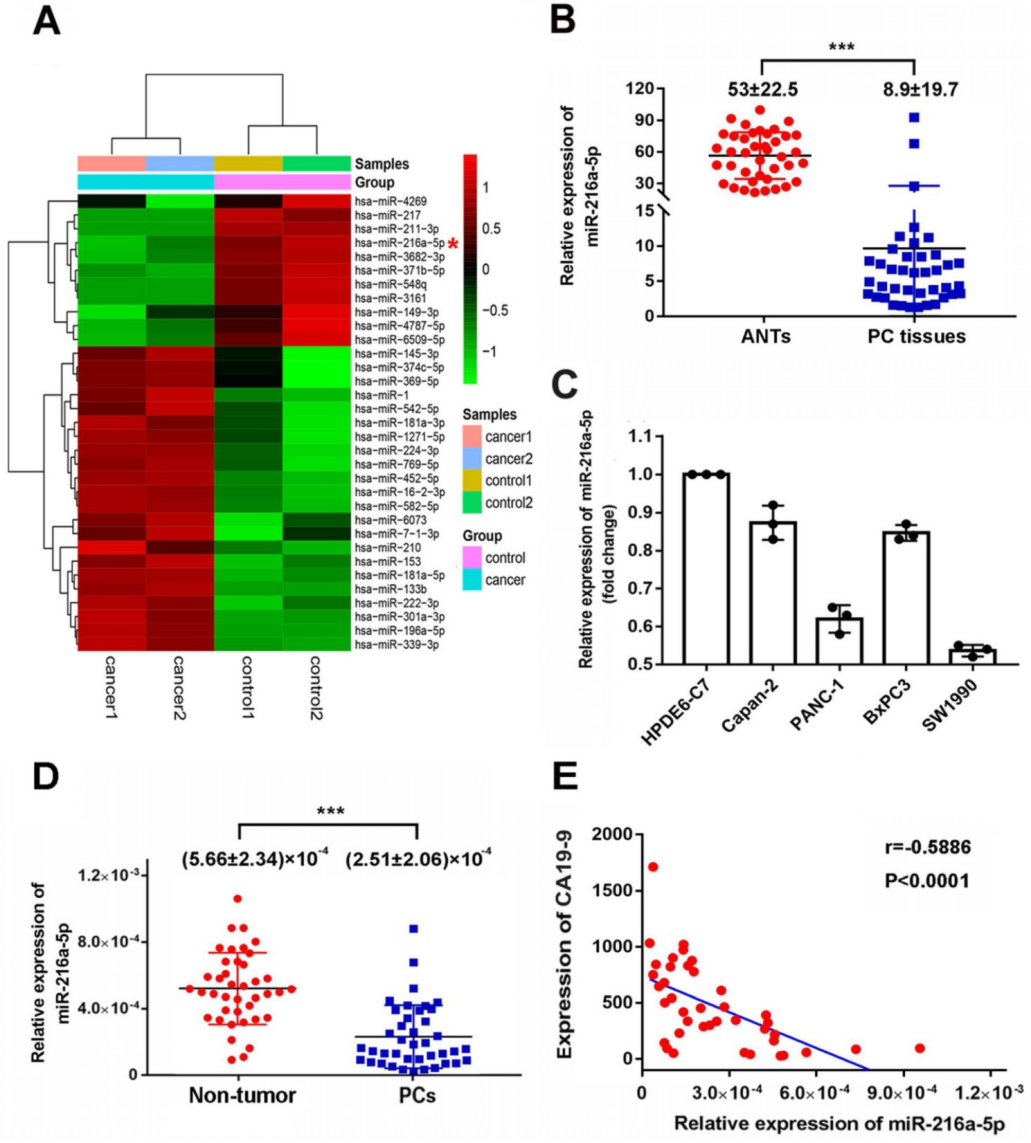
** Expression levels of miR-216a-5p in PC tissues and plasma specimens.** (**A**) Heat map showing differential miRNAs expression of ANTs and PC tissues examined by microarray. (**B**) Expression of miR-216a-5p in 40 pairs of PC tissues were assessed by qRT-PCR and normalized to U6 expression. The miR-216a-5p level in PC tissues and ANTs were compared using paired Student's *t*-test. (**C**) Relative expression levels of miR-216a-5p in four PC cell lines were determined with qRT-PCR. (**D**) Expression levels of miR-216a-5p in the plasma of 40 pairs of PCs patients and patients without pancreatic disease. Between-group differences were assessed using the unpaired Student's *t*-test. (**E**) Negative correlation between miR-216a-5p and CA19-9 levels in the plasma of PC patients (n=40); ***P<0.001.

MicroRNA array was firstly used to compare the miRNAs expression patterns between PC tissues and paired ANTs. MiR-216a-5p was found to be significantly downregulated by 6.18-fold in PCs compared to the paired ANTs (Fig. 1A). Real-time PCR was further performed to measure the expression level of miR-216a -5p in 40 pairs of PC tissues and ANTs. Consistent with the sequencing data, the expression of miR-216a-5p in PC tissues was significantly decreased as compared to that in ANTs (Fig. 1B). Moreover, we examined the expression of miR-216a-5p in four PC cell lines along with one normal pancreatic ductal epithelial cell line (HPDE6), miR-216a-5p was found significantly down-regulated in all PC cells including PANC1, Capan-2, BxPC-3, and SW1990, as compared with HPDE6. Among these, SW1990 showed the lowest level of miR-216a -5p, while Capan-2 and BxPC3 exhibited higher level as compared to SW1990 and PANC-1 (Fig. 1C).

To evaluate the correlation between miR-216a-5p and clinicopathological parameters, forty PCs patients were divided into two groups according to the expression level of miR-216a-5p in tumor tissues. The median miR-216a-5p level was regarded as the cut-off point for low and high expression (0.620132, as determined by qRT-PCR when normalized to U6 RNA as the endogenous control). As indicated in Table 1, the level of miR-216a-5p was negatively associated with PCs peripancreatic lymphatic metastasis (*p*=0.0285), perineural invasion (*p*=0.001) and advanced TNM stage (*p*<0.001). Lower expression of miR-216a-5p usually predicted a worse TNM stage (III and IV) and advanced progression, while higher miR-216a-5p expression was associated with less tumor metastasis and lower TNM stage (II). There were no statistical correlations between miR-216a-5p expression and gender, tumor size, differentiation or angiolymphatic invasion in PC patients.

We also examined the miR-216a-5p levels in circulating plasma of PC patients. As expected, PC patients showed significantly lower level of plasma miR-216a-5p as compared with normal volunteers (Fig. 1D). Noting CA19-9 is commonly used as a diagnostic marker for PCs, here, we analyzed the correlation between CA19-9 and miR-216a-5p. The results showed a negative correlation between the plasma miR-216a-5p and the corresponding CA19-9 levels (Fig. 1E). All above results indicate that downregulation of miR-216a-5p may contribute to tumor malignancy and can be used as a potential marker to predict PC.

### MiR-216a-5p suppresses the PC cells proliferation and migration* in vitro*

**Figure 2:**
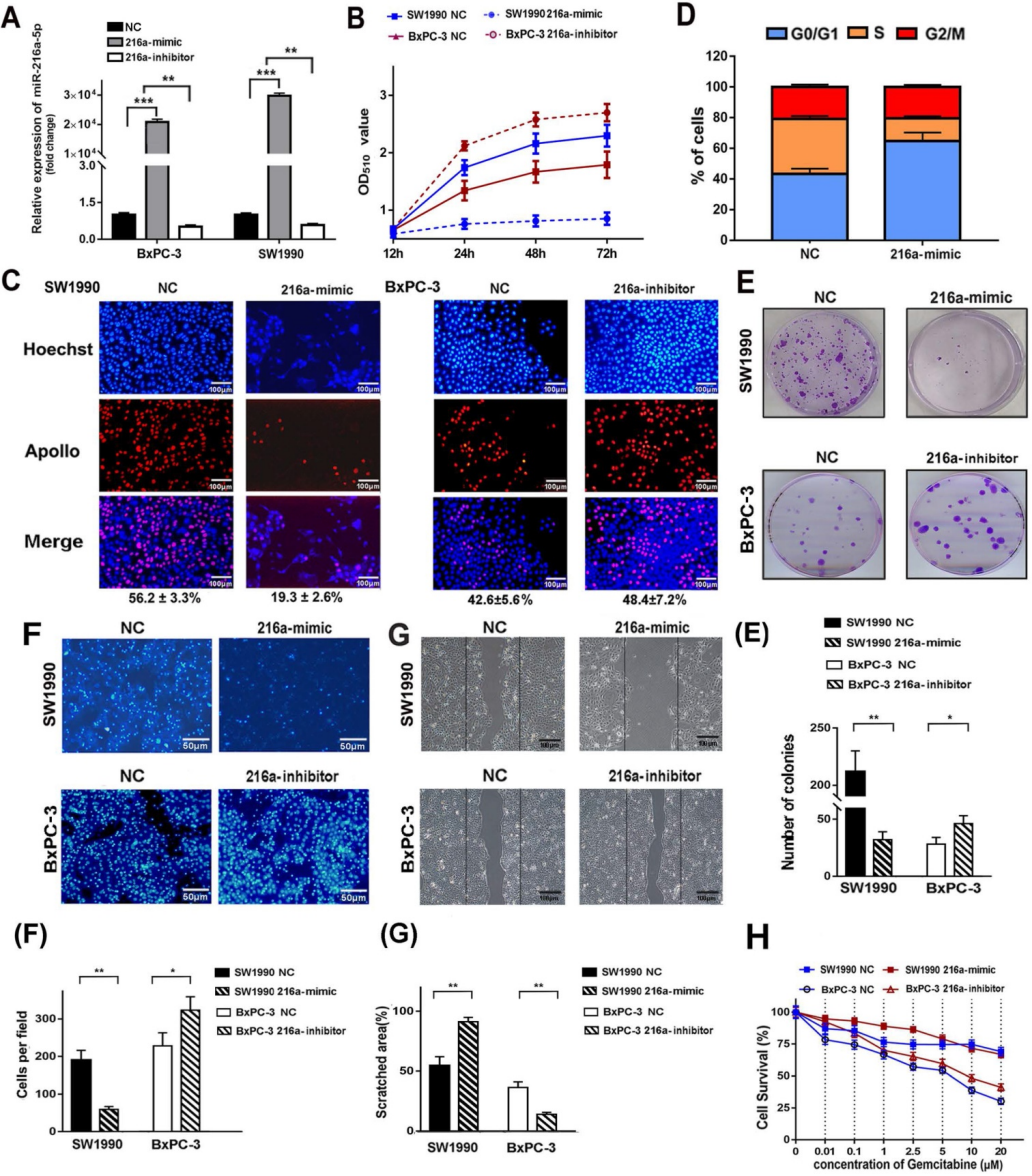
** Effects of miR-216a-5p on PC cell proliferation and migration *in vitro*.** SW1990 and/or BxPC-3 were transfected with miR-216a-5p mimic, miR-216a-5p inhibitor or their corresponded negative controls, respectively. (**A**) Overexpression of miR-216a-5p was determined by qRT-PCR in both cell lines. (**B**) Cell growth was examined with SRB analysis at different time points (12, 24, 48, and 72h). (**C**) Cell cycle distribution of SW1990 cells was determined by PI staining and flow cytometry analysis. (**D**) Cell proliferation was analyzed by EDU incorporation assay on the 3^rd^ day after transfection. Scale bar, 100 µm. (**E**) Colony forming assays was performed to detect the colony formation ability of SW1990 cells after transfection with miR-216a-5p. (**F**) Transwell invasion assays and (**G**) wound healing assays were performed. Scale bars, 50μm and 100 µm respectively. The invaded cells and scratched areas were photographed at the indicated time-point. The number of migrated cells in transwell chambers and the percentage of scratched area were compared in the diagram. (**H**) The viability of both cell lines exposed to Gemcitabine was determined by SRB analysis after transfection with miR-216a-5p mimic. Results presented as mean (±SD) of three independent experiments. *P<0.05; **P<0.01; ***P<0.001.

To investigate the biological function of miR-216a-5p in PC cells, SW1990 and BxPC-3 cells were transfected with miR-216a-5p mimic or inhibitor respectively. Real-time PCR data revealed significant upregulation of miR-216a-5p in SW1990 and downregulation in BxPC-3 cells (Fig. 2A). Functionally, SRB analysis showed that miR-216a-5p mimic significantly inhibited cell growth in SW1990 cells, and miR-216a-5p inhibitor significantly promoted cell growth in BXPC-3 cells as compared with negative control (NC) (Fig. 2B). EdU incorporation assay further confirmed that cell proliferation was apparently inhibited by miR-216a-5p mimic, whereas promoted by miR-216a-5p inhibitor, with visible decreased or increased EdU incorporation in both cell lines (Fig. 2C). Cell cycle analysis demonstrated that miR-216a-5p mimic caused significant accumulation of cells in G0/G1 phases and a reduction in S phase in SW1990 cells, whereas, miR-216a-5p inhibitor increased cells in S phase and reduced in G0/G1 phases in BxPC-3 cells (Fig. 2D). Moreover, miR-216a-5p mimic remarkably inhibited the colony formation in SW1990 cells, and as expected, miR-216a-5p inhibitor enhanced colony formation in BxPC-3 cells (Fig. 2E).

Wound healing and Transwell assay was carried out to explore the role of miR-216a-5p on cell metastasis and invasion. As indicated in Fig. 2F and G, miR-216a-5p mimic significantly inhibited the wound closure and the migration ability of SW1990 cells; In contrast, knockdown of miR-216a-5p accelerated wound closure and migration ability of BXPC-3 cells. Additionally, we also found miR-216a-5p mimic significantly improved the sensitivity of SW1990 cells to gemcitabine, a first-line chemotherapy drug for PC; however, miR-216a-5p inhibitor enhanced the resistance in BxPC-3 cells (Fig. 2G). Collectively, these results demonstrate that miR-216a-5p is an important tumor suppressor in PCs, which effectively suppress cell proliferation and migration *in vitro.*

### MiR-216a-5p suppresses tumor growth and metastasis of PCs *in vivo*

**Figure 3:**
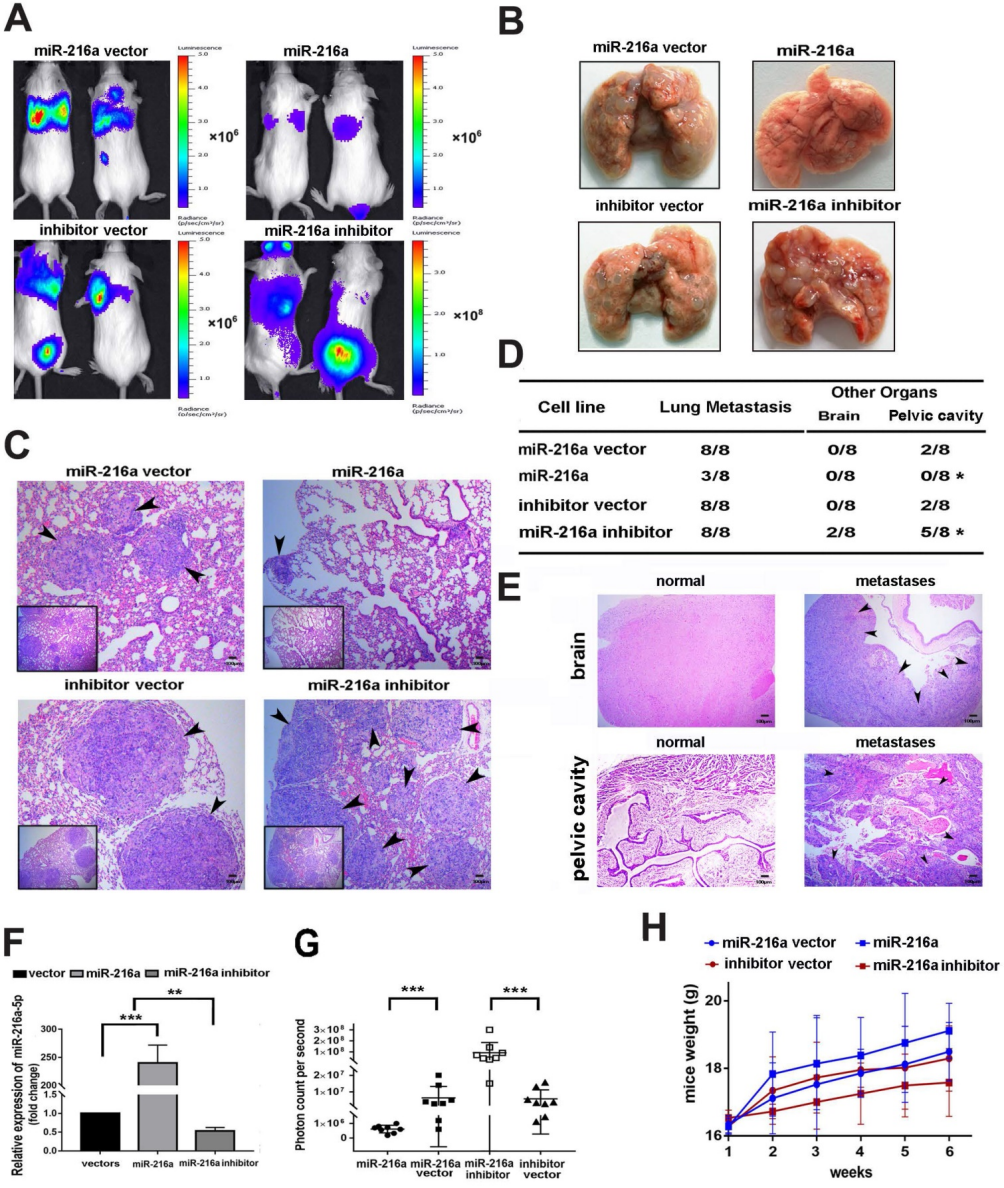
** Effects of miR-216a-5p on PC cell proliferation and metastasis *in vivo*.** (**A**) Indicated cells were injected into SCID mice through tail vein. The tumor burden was detected and presented with the bioluminescence images. (**B**) Mice were sacrificed and the lung tissues are presented (**C**) Lung metastases of PC mice model were confirmed by H&E staining. Arrows indicate tumor lesions. Scale bar, 100 µm. (**D**) The incidence of metastasis in brain or pelvic cavity in each group. (**E**) Brain and pelvic cavity metastases lesions were evaluated by H&E staining. Scale bar, 100 µm. (**F**) The miR-216a-5p expressions in each group were determined by qRT-PCR. (**G**) The photon counts per second were recorded to assess the metastatic mass (***P < 0.001). (**H**) The weight of SCID mice was measured once a week for a total of 6 weeks.

To clarify the inhibitory efficacy of miR-216a-5p* in vivo*, SW1990 was chosen to establish the lung metastasis model by tail vein injection, considering it is more malignant and have very low background of miR-216a-5p, which is much easier to elucidate the inhibitory efficacy of miR-216a-5p mimic. As shown in Fig. 3A and B, miR-216a-luc2-transduced SW1990 cells showed significantly less and smaller pulmonary colonization, about 2mm in diameter, than the vector control that were 4-5mm in diameter, and only very weak luciferase activity was observed, which pathology was further confirmed by H&E staining analysis (Fig. 3C). Importantly, the lung seeding activity of SW1990 could be enhanced after knock-down of the limited endogenous miR-216-5p (Fig. 3A-C). Meanwhile, the tumor burden level was significantly decreased in mice inoculated with miR-216a-luc2-transduced SW1990 cells, but was increased for miR-216^-KD^ SW1990 cells (Fig. 3D). Brain and pelvic cavity metastases were examined by histological analyses (Fig. 3E). Certainly, to confirm the efficacy of stably transduced cells* in vivo*, we examined the expression level of miR-216a-5p in lung metastases by randomly selecting 4 mice from 8 in each group. As expected, miR-216a-5p expression was significantly increased in SW1990-216a-*luc*2 cells, whereas, decreased in SW1990-216a^-KD^-*luc*2 cells generated lung metastasis tissues, compared with their respective NC groups (Fig. 3F). Moreover, bioluminescence assay was performed to demonstrate metastases in each mouse in all four groups, and the results were consistent with the above results. The observed bioluminescence was very low in miR-216a mimic-transduced group (approximately 10^6^ photon count/sec); however, it was very high in miR-216a-5p^-KD^ transduced group (approximately 10^8^ photon count/sec) (Fig. 3G). In addition, the body weight in miR-216a group was distinctly better than that in NC group mice, especially as compared to that in miR-216a^-KD^ group (Fig. 3H). Taken together, these results demonstrate that miR-216a-5p effectively reduced tumor progression of PC *in vivo*.

### TPT1 is a direct target of miR-216a-5p

**Figure 4:**
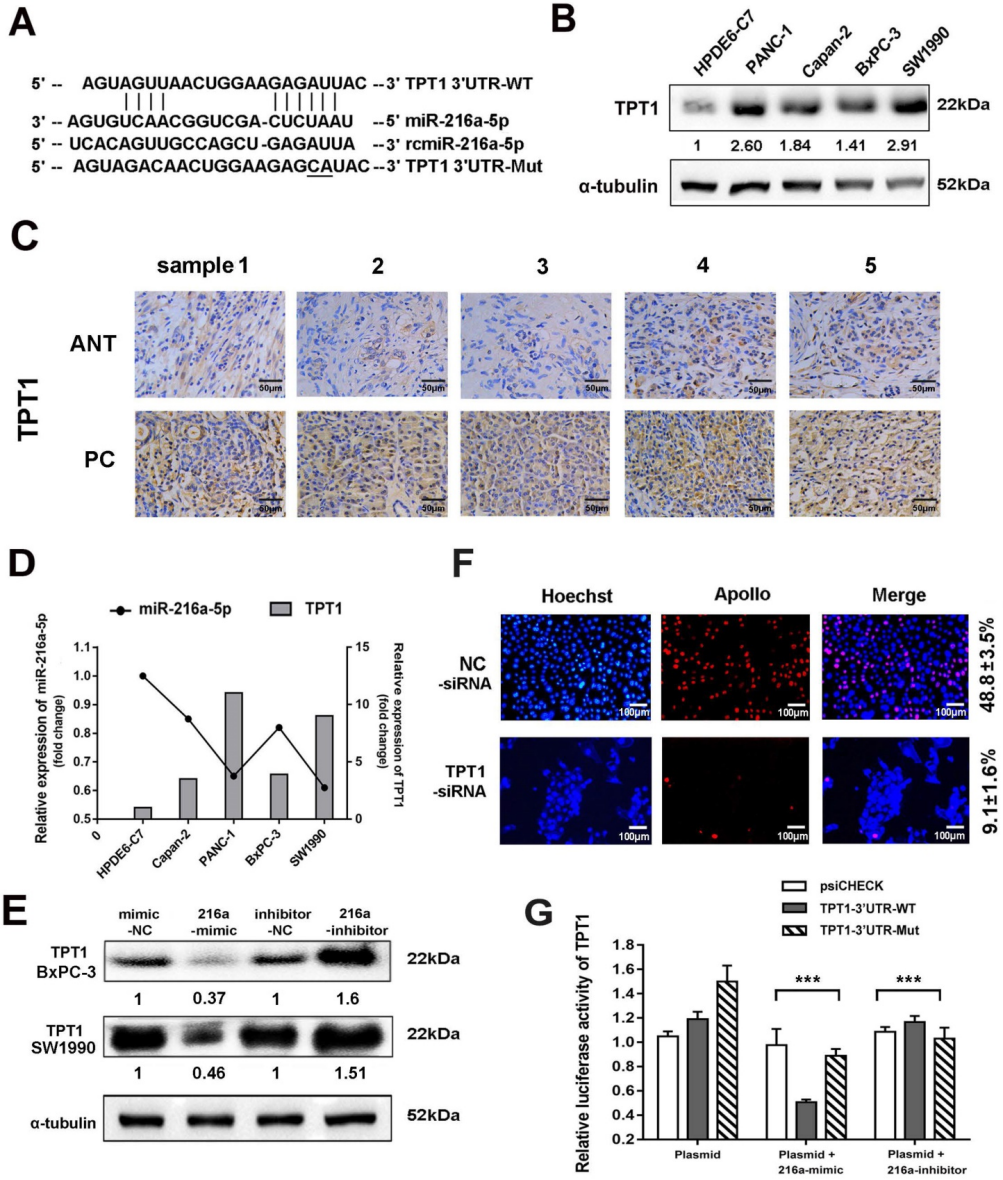
** TPT1 is a downstream target of miR-216a-5p.** (**A**) Schematic illustration of the two predicted binding sites and mutation sequence between miR-216a-5p and TPT1. (**B**) Western blot were performed to evaluate the expression of TPT1 in five cell lines. (**C**) Expression of TPT1 in PCs and ANT tissues were detected by IHC staining (40×). Scale bar, 50 µm. (**D**) Relationship between miR-216a-5p and TPT1 was analyzed in different cell lines. (**E**) TPT1 expressions in SW1990 and BxPC-3 were examined by Western-blot after transfection with miR-216a-5p mimics and inhibitor. (**F**) Cell proliferation after TPT1 knockdown was assessed using EdU assay. Scale bar, 100 µm. (**G**) The luciferase activities of TPT1 were measured using the dual-Luciferase reporter assay after transfection of reporter plasmid alone or in the presence of miR-216a-5p mimic or inhibitor. Data are representative of three experiments. Error bars represent ±SD. ***P < 0.001 vs. vector alone group.

Two programs (Target Scan and microRNA.org) were applied to predict the potential target genes of miR-216a-5p. TPT1 was predicted as the candidate target of miR-216a-5p (Fig. 4A), which has been reported to contribute to tumor progression such as hepatocellular carcinoma, lung cancer and prostate cancer 17. Herein, TPT1 was observed significantly overexpressed in both PCs cell lines and tissues as compared to the pancreatic ductal epithelial cells and ANTs, through western blot and immunohistochemical (IHC) staining (Fig. 4B and C). The IHC results also indicated that TPT1 was mainly localized in the cytoplasm, although weak immunoreactivity was visible in the nucleus. We then examined the correlation between miR-216a-5p and TPT1 expression and found that the level of TPT1 expression was inversely correlated with miR-216a-5p expression in all four PC cell lines (Fig. 4D). Importantly, transfection of SW1990 cells with miR-216a-5p mimic or inhibitor dramatically suppressed or enhanced expression of TPT1 compared with the negative control (Fig. 4E). As expected, knockdown TPT1 by its specific siRNA significantly suppress the proliferation of cells (Fig. 4F).

To further confirm TPT1 is a direct target of miR-216a-5p, luciferase reporter vectors containing theoretical seed sequences in the 3'-UTR of TPT1 (wild-type or mutant) were constructed and transfected in HEK-293T cells, either alone or in combination with miR-216a-5p mimic/inhibitor. As shown in Fig. 4G, miR-216a-5p mimic remarkably suppressed, whereas miR-216a-5p inhibitor upregulated the activity of RLuc involving the seed sequence in the 3'-UTR of TPT1 in HEK293T cells. Meanwhile, the mutant vectors involving the TPT1 3'-UTR binding site abolished these effects on RLuc activity. These data indicate that miR-216a-5p can directly target TPT1 and suppress its expression.

### TPT1 is essential for miR-216a-5p-mediated tumor progression via regulation of mTORC1 signaling

**Figure 5:**
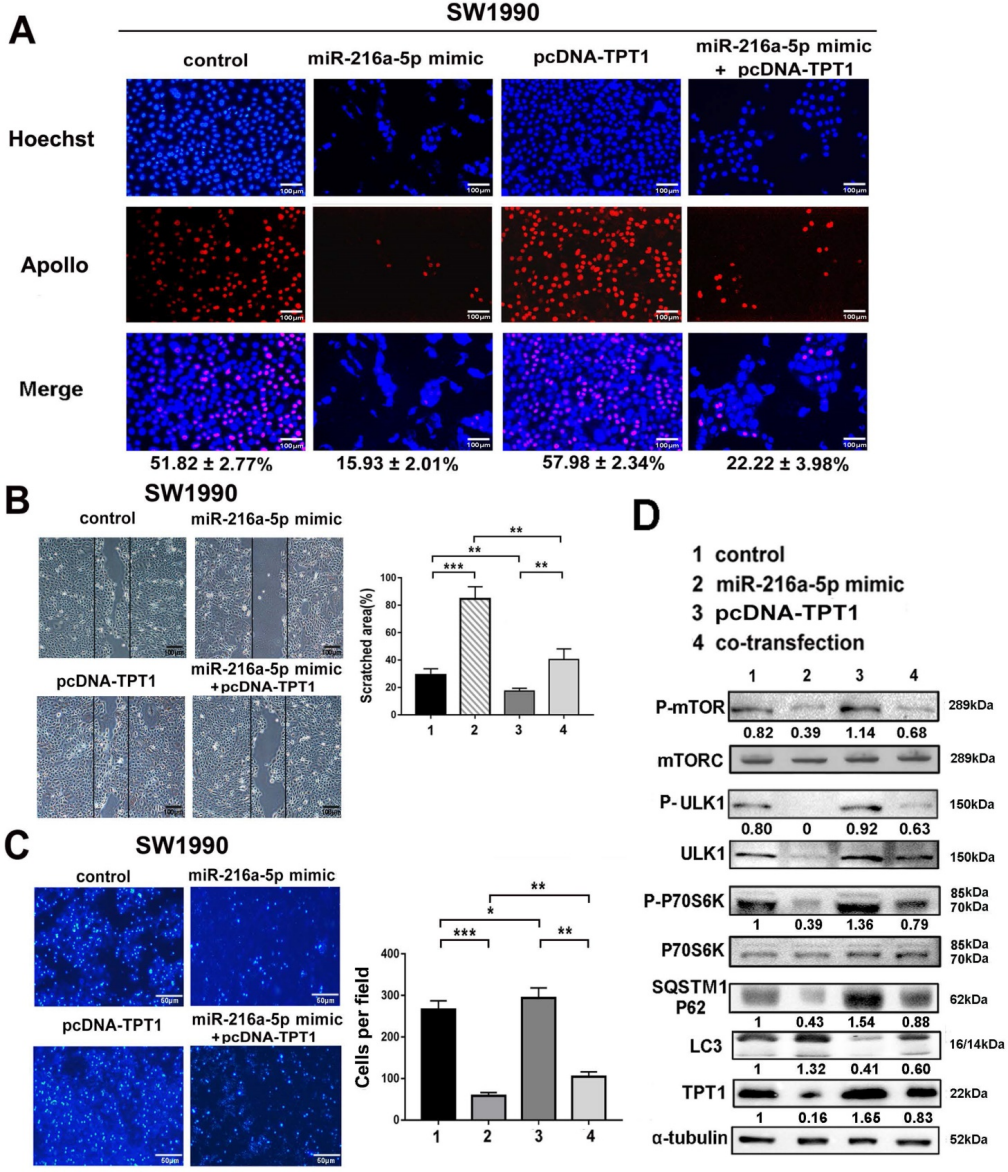
** TPT1 contributes to miR-216a-5p-mediated malignance through mTORC1 pathway.** (**A**) EdU incorporation assay, (**B**) wound healing assay and (**C**) Transwell migration assay were carried out to detect the effects of TPT1 and miR-216a-5p on cell proliferation and migration. Scale bars, 100 µm, 100 µm and 50 µm respectively. The number of stained cells and percentage of scratched areas were measured and compared at 24h in the diagrams. (**D**) Western blot was conducted to detect the related pathways proteins of TPT1.

To determine whether miR-216a-5p-mediated tumor growth and metastasis is regulated by TPT1, gain- and loss-of-function experiments were performed using miR-216a-5p mimic and TPT1 expression plasmid lacking 3'-UTR. As demonstrated in Fig. 5A-C, overexpression of TPT1 effectively attenuated miR-216a-5p mimic-mediated inhibition of cell proliferation, wound closure and transwell abilities in SW1990. Meanwhile, we noticed that overexpression of TPT1 could not completely compensate for the inhibitory effects of miR-216a-5p mimic, which implied some other targets of miR-216a-5p may also contribute to PCs tumor progression, and here we found TPT1 is an important one.

Previous studies have revealed that TPT1 may participate in tumor malignancy *via* several intracellular signaling pathways. In this study, we examined several possible pathway proteins that might be regulated by TPT1. As demonstrated in Fig. 5D, exogenous TPT1 induced the phosphorylation of mTORC1, ULK1 (downstream kinase that can be phosphorylationally-activated by mTOR at Ser757) and P70S6K (direct downstream signaling molecule of mTORC1 that can be phosphorylationally-activated at Try229 and 389), subsequently, with decreased LC3 (the autophagy marker) and increased SQSTM1/p62 (the autophagy substrate). This indicated that TPT1-induced tumor progression is at least partially attributable to its suppressive effect on mTOR-dependent autophagy. Importantly, miR-216a-5p mimic had the converse effect compared with TPT1 plasmid; furthermore, when cells received both miR-216a-5p mimic and TPT1 plasmid treatment, the effects were neutralized by each other. These data demonstrate that miR-216a-5p mediate tumor inhibition by targeting TPT1 and TPT1-mediated autophagy is likely involved in PCs progression.

### LncRNA01133 and miR-216a-5p interact with and repress each other

**Figure 6:**
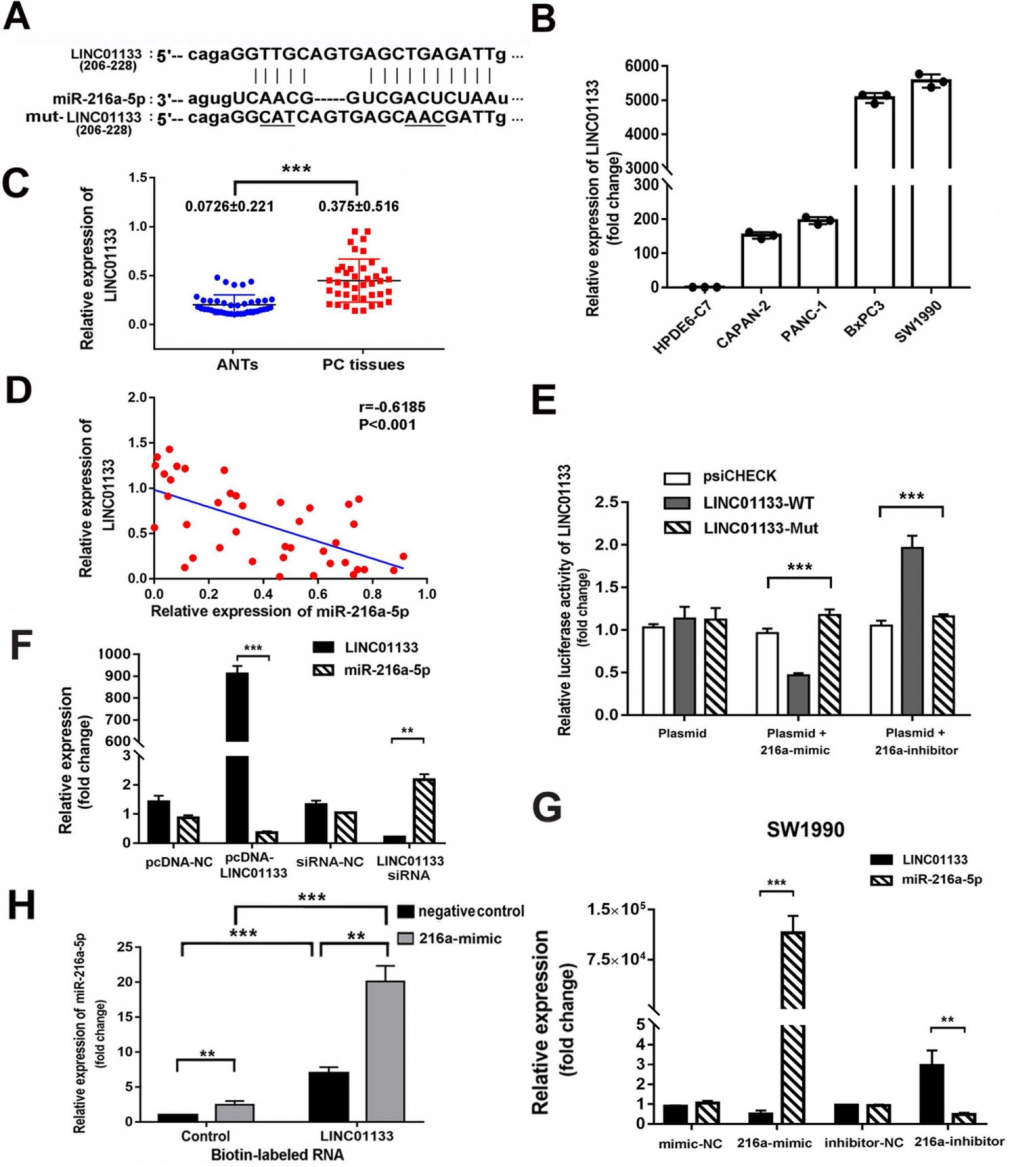
** Reciprocal repression between LINC01133 and miR-216a-5p.** (**A**) Schematic illustration of the miR-216a-5p predicted binding sites and the corresponding site in LINC01133 mRNA sequence. (**B**) and (**C**) Relative LINC01133 expression in different cell lines and in 40 different pairs of PC tissues was examined by qRT-PCR. Data were normalized to U6 RNA as an endogenous control. (**D**) Pearson correlation analysis shows the negative relationship between LINC01133 and miR-216a-5p in PC tissues. (**E**) The luciferase activities of LINC01133 were measured using the luciferase reporter assay after transfection of HEK293 cells alone or in the presence of miR-216a-5p mimic or inhibitor. (**F**) and (**G**) Relative expressions of miR-216a-5p and LINC01133 were measured by qRT-PCR after cells were transfected with pcDNA-LINC01133, miR-216a-5p mimic/inhibitor, or its corresponding control. (**H**) The level of miR-216a-5p was detected by q-PCR in indicated cell lysates that pulled down by biotin-labeled LINC01133 or control RNA. All data are representative of three independent experiments. Error bars represent ±SD. **P < 0.01, ***P < 0.001.

Recently, lncRNAs have been reported as ceRNA which communicate with and regulate each other by sponging miRNAs and in turn regulate the expression of target genes. To assess whether miR-216a-5p is regulated by lncRNA, two bioinformatics databases (Miranda and Starbase v.2.0) were used to predict their interaction sites. LINC01133, which is transcribed from the 1 chromosome, was predicted to harbor two conserved binding sites for miR-216a-5p (Fig. 6A). Real time PCR results revealed LINC01133 was significantly upregulated in PC cell lines as compared with normal cell line. Similar results were observed in forty PC tissues and corresponding ANTs (Fig. 6B, C). Pearson correlation analysis further revealed a negative association between miR-216a-5p and LINC01133 expression in PC tissues (r = -0.6185, *P* < 0.001, Fig. 6D).

To determine whether the direct binding occurs between LINC01133 and miR-216a-5p, luciferase reporter vectors containing the theoretical seed sequences in 3'-UTR of LINC01133 and the corresponding mutant vectors were constructed for transfection of HEK293T cells respectively, either alone or in combination with miR-216a-5p mimic or inhibitor. Luciferase activity indicated that miR-216a-5p mimic significantly suppressed, whereas its inhibitor promoted the Rluc activity that carried LINC01133-wild type (WT) but not with the mutant (Mut) vectors (Fig. 6E). Moreover, qRT-PCR results showed that miR-216a-5p expression was depressed by exogenous LINC01133, but upregulated by LINC01133-siRNA in SW1990. Intriguingly, LINC01133 expression was downregulated by miR-216a-5p mimic, whereas, upregulated by miR-216a-5p inhibitor in SW1990 cells (Fig. 6F, G). Importantly, we further employed the biotinylation labeled pulldown system to confirm the direct binding between LINC01133 and miR-216a-5p. Biotin-labeled control RNA or LINC01133 were incubated with SW1990 cell lysates in which transfected negative control or miR-216a-5p mimic respectively. Measured by qPCR, a significant amount of binding miR-216a-5p was observed in the LINC01133 pulled down pellet compare with control (P<0.01, Fig. 6H). The FISH data indicated that the majority of LINC01133 was distributed in nuclei while the rest located in cytoplasm (Fig. S1A), this was further confirmed by nuclear/cytoplasmic fractionation assay (Fig. S1B). Collectively, these results demonstrate the reciprocal repression between LINC01133 and miR-216a-5p, and LINC01133 may act as a sponge of miR-216a-5p in PCs.

### LINC01133 promotes cell proliferation and metastasis by regulating miR-216a-5p/TPT1 in PCs

**Figure 7:**
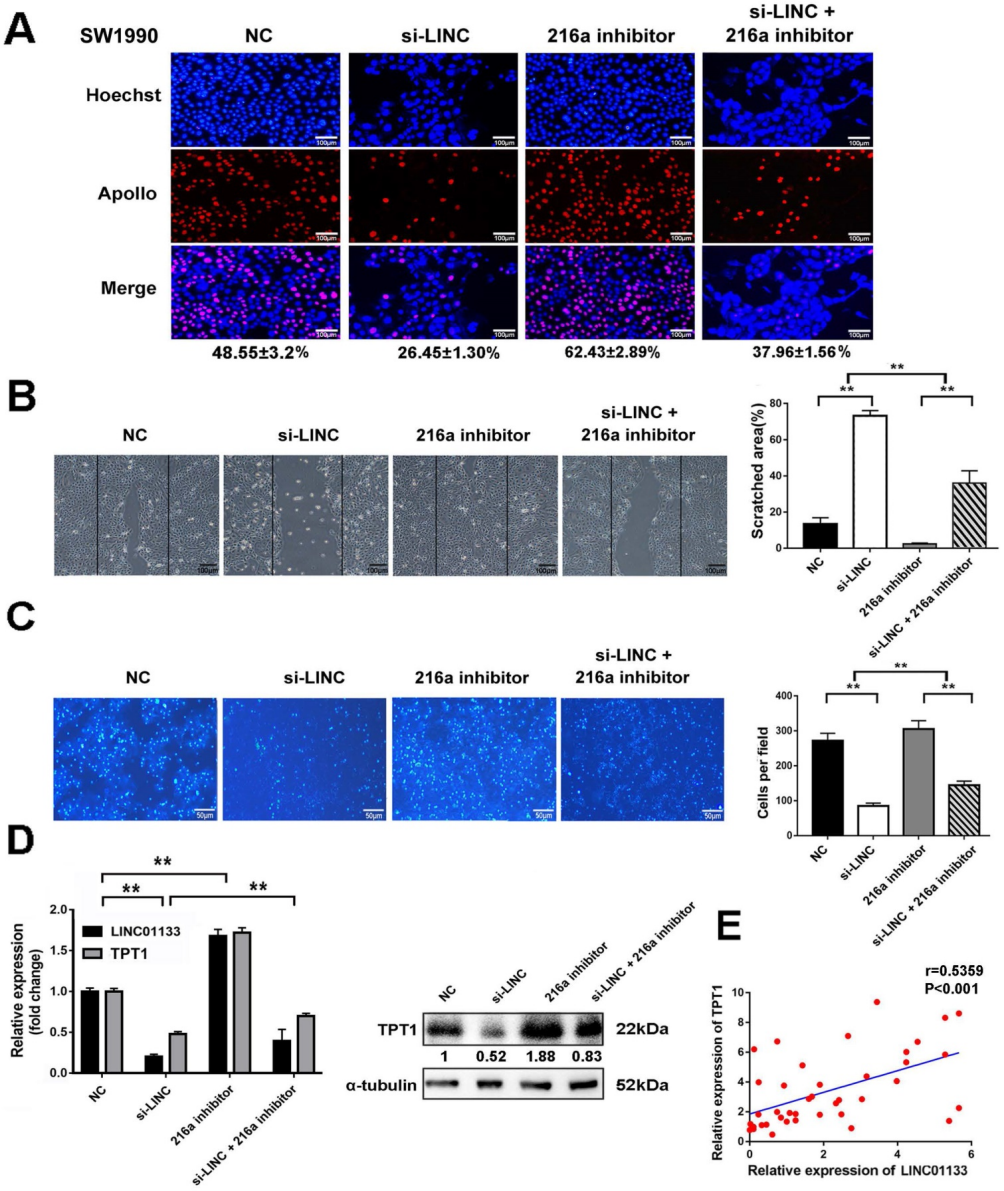
** LINC01133 promotes cell proliferation and metastasis by regulating miR-216a-5p/ TPT1 in PCs.** LINC01133-siRNA and pcDNA-TPT1 were transfected into SW1990 cells alone or in combination. (**A**) EdU incorporation assay, (**B**) wound healing assay and (**C**) transwell migration assay were performed. Scale bars, 100 µm, 100 µm and 50 µm respectively. (**D**) The expression of LINC01133 and TPT1 were detected by qRT-PCR after cells received indicated transfection. The expression of TPT1 proteins were detected by Western blot. (**E**) Relative expression of LINC01133 and TPT1 in 40 different pairs of ANT and PC tissues were examined by qRT-PCR.

To clarify that LINC01133 promotes malignancy by regulating miR-216a-5p/TPT1 in PC. LINC01133 was knocked down by its specific siRNA (si-LINC). The results demonstrated that si-LINC transfection significantly inhibited cell proliferation, invasion and migration in SW1990 cells. Intriguingly, miR-216a-5p inhibitor moderately abolished the suppressive effect of LINC01133-knockdown on the malignant attributes of cells (Fig. 7A-C). Importantly, TPT1 expression could be inhibited by si-LINC01133, and this inhibitory effect was abolished by miR-216a-5p inhibitor (Fig. 7D). Meanwhile, Pearson correlation analysis further revealed a positive association between TPT1 and LINC01133 expression in PC tissues (r=0.5359, *P*<0.001, Fig. 7E). Taken together, our data validated that miR-216a-5p/TPT1-mediated tumor malignancy can be regulated by LINC01133 in PCs.

## Discussion

The lack of valid biomarkers is the main bottleneck that preventing appropriate diagnosis and effective treatment of PC. Accordingly, PC associated survival rates are as low as 9%, and for patients diagnosed at an advanced stage, the survival rates are only 2% 1. Molecular aspects such as the key genes responsible for driving PC progression have been identified. However, identification of novel biomarkers for early diagnosis and development of effective therapies are still on the way to curb the aggressive behavior of PC.

Increasing body of evidence has implicated the dysregulation of non-coding RNAs in the genesis and progression of all types of cancers. However, fewer studies have reported the roles of miR-216a in tumor progression and the associated mechanisms, even so, the opposite functions of miR-216a-5p were found in limited reported. For example, miR-216a-5p was shown to contribute to tumor regression by targeting PAK2 or Bcl-2 family proteins in breast and small cell lung cancers 18. lncRNA HCP5 was shown to promote the progression of follicular thyroid carcinoma by acting as a sponge for miR-186-5p and miR-216a-5p 19. Contradictorily, miR-216a-5p was found to be an oncogene in renal cell carcinoma 8. However, the role and the regulatory mechanism of miR-216a-5p in PC are not well characterized.

In this study, we observed significant downregulation of miR-216a-5p in PC tissues; in addition, the expression level of miR-216a-5p was a negatively correlation with clinical parameters of PC, including TNM stage, peripancreatic lymphatic metastasis, and perineural invasion. We also found that the plasma miR-216a-5p level was usually decreased in PC patients compared with normal persons, which presents a positive correlation with the corresponding CA19-9 level (Fig. 1E), thereby implying that circulating miR-216a-5p may be a potential clinical marker in the context of PC.

Furthermore, we found that miR-216a-5p effectively inhibited tumor proliferation and metastasis both *in vitro* and *in vivo*, which plays a crucial role in PC progression. Bioinformatics analysis and luciferase reporter system showed that TPT1 is an important target of miR-216a-5p. Our results also showed an inverse correlation between TPT1 and miR-216a-5p expression levels in PC tissues and cells. Moreover, the exogenous expression of TPT1 effectively attenuated the inhibitory effect on cell proliferation and metastasis, which was induced by miR-216a-5p mimic. This indicates that miR-216a-5p effectively suppressed the progression of PC *via* downregulating its pivotal target TPT1.

Noting the expression of TPT1 was not completely consistent with the level of miR-216a-5p (Fig. 4). We hypothesized whether ubiquitination could contribute to the rapid degradation of TPT1, after detecting the half-life of TPT1, we confirmed that TPT1 is very stable with a half-life of > 48 hrs, thus TPT1 expression may have been influenced by multiple factors: transcriptional factors, post-transcriptional modification, and more miRNAs. The feedback loop has also been reported between TPT1 and p53, wherein p53 leads to transcriptional repression of TPT1 by directly interacting with its promoter, and TPT1 promotes MDM2-dependent degradation of p53 20. As p53 is frequently mutated in tumor cells, including PC cell lines SW1990 and PANC-1, p53 would not be a primary regulator of TPT1 in these cell lines. Recently, TPT1 was found to be regulated by non-coding RNAs, such as miR-145-5p, which improved drug resistance by controlling TPT1 in prolactinoma 15. lncRNA-TPT1-AS1 promotes tumor progression by inducing TPT1 and downstream PI3K/AKT signaling in epithelial ovarian cancer 21. lncBRM/miR-204-3p/TPT1 and LINC01446/miR-489-3p/TPT1 axis participate in tumor progression in colorectal cancer and glioblastoma 22. Notably, as revealed in our study, miR-216a-5p and LINC01133 may play an important role in regulating the expression of TPT1 in PCs.

Amounts of miRNAs have been found to be regulated by lncRNAs. LncRNA DANCR, lncRNA A1BG-AS1, and lncRNA HOTTIP were found to contribute to tumor progression by interacting with miR-216a-5p in breast cancer, hepatocellular carcinoma and prostate cancer respectively 23. Through analyzing the databases GSE16515 and GSE32688, LINC01133 was predicted to act as a sponge for miR-216a-5p. LINC01133 plays opposite roles in several types of cancers, as either an oncogene or tumor suppressor. In previous studies, C/EBPβ-LINC01133 axis was found to promote proliferation of pancreatic ductal adenocarcinoma cells by upregulating CCNG1 24. In addition, LINC01133 was found to aggravate the progression of hepatocellular carcinoma and non-small cell lung cancer by activating the PI3K/ATK pathway or by repressing KLF2/E-cadherin 25, 26. Conversely, LINC01133 was reported to act as a tumor suppressor in the context of gastric cancer, which was downregulated in tumor cells and acts as a ceRNA by sponging miR-106a-3p to regulate APC expression and Wnt/β-catenin pathway 27, 28. It was reported to inhibit EMT in colorectal cancer by interacting with SRSF6 29 and to inhibit tumor metastasis through a feedback regulation loop with GDF15 in oral squamous cell carcinoma 30. In this study, we identified that LINC01133 was upregulated in PCs. Although the majority of LINC01133 focused in nuclear, the minority which distribute in cytoplasm could exert potential onco-function by interacting with miR-216a-5p with each repressing the expression of the other.

In summary, our results demonstrated that miR-216a-5p is significantly downregulated in PC tissues and plasma. Decreased expression of miR-216a-5p promotes PC cell growth and progression both *in vitro* and *in vivo*. TPT1 was one of the direct targets of miR-216a-5p and contributed to miR-216-5p-induced tumor malignancy *via* mTOR-dependent autophagy. Moreover, LINC01133 was found to promote tumor proliferation and metastasis by suppressing the expression of miR-216a-5p in PC. Our study not only revealed the important role of LINC01133/miR-216a-5p/TPT1 signaling pathway in the pathogenesis of PCs but also indicates a potential role of miR-216a-5p and LINC01133 in the clinical diagnosis and therapy of PCs.

## Supplementary Material

Supplementary figure S1.

## Figures and Tables

**Table 1:** The relationship between clinicopathological parameters and miR-216a-5p expression in 40 Pancreatic Cancers

Variable	Number of patients	Expression level of miR-216a-5p		*P*-value
		low^#^	high ^#^	
Overall	40	20	20	
**Age**				0.011 *
≥ 60	20	14	6	
< 60	20	6	14	
**Gender**				0.723
Male	29	15	14	
Female	11	5	6	
**Tumor size**				0.705
≥ 30 mm	31	15	16	
< 30 mm	9	5	4	
**Differentiation**				1.000
Well	0	0	0	
Moderate	32	16	16	
Poor	8	4	4	
**Peripancreatic lymph**				0.0285*
Negative	10	2	8	
Positive	30	18	12	
**TNM stage ^##^**				0.000**
I/II	19	3	16	
III/V	21	17	4	
**Perineural invasion**				0.001**
Negative	18	4	14	
Positive	22	16	6	
**Angiolymphatic invasion**				0.429
Negative	8	3	5	
Positive	32	17	15	

#. Median miR-216a-5p level is used as the cut-off, which is 0.620132, according to the result of qRT-PCR that normalized to U6. ##. P-values of χ2-test are shown. * P < 0.05; ** P < 0.01.

## Abbreviations

ANT: adjacent normal tissue
ceRNA: competing endogenous RNA
DAPI: 4',6-diamidino-2-phenylindole
EMT: Epithelial-Mesenchymal Transition
FBS: fetal bovine serum
IHC: immunohistochemistry
LINC01133: long intergenic none-protein coding RNA 1133
miRNA: microRNA
mTOR: mammalian target of rapamycin
NC: negative control
PAK2: p21 activated kinase 2
PC: pancreatic cancer
PI: 4',6-diamidino-2-phenylindole
PI3K: phosphatidylinositol 3 kinase
qPCR: quantitative polymerase chain reaction
SCID: severe combined immunodeficient
SRB: sulforhodamine B
TCA: trichloroacetic acid
TPT1: tumor protein translationally controlled 1
WT: wild type
