# MiR-216a-5p inhibits tumorigenesis in Pancreatic Cancer by targeting TPT1/mTORC1 and is mediated by LINC01133: Erratum

**DOI:** 10.7150/ijbs.70911

**Published:** 2022-03-03

**Authors:** Jian Zhang, Shuohui Gao, Yandong Zhang, Huixin Yi, Mengxian Xu, Jialun Xu, Huan Liu, Zhichen Ding, Hongbin He, Hongmei Wang, Zhuo Hao, Liankun Sun, Yan Liu, Feng Wei

**Affiliations:** 1 Department of Hepatobiliary and Pancreas Surgery, Jilin University First Hospital, Changchun, China.; 2 Genetic Engineering Laboratory of PLA, Institute of Military Veterinary Medicine, Academy of Military Medical Sciences, Changchun, China.; 3 Department of Gastrointestinal Colorectal Surgery, China-Japan Union hospital of Jilin University, Changchun, China.; 4 Department of Pathophysiology, College of Basic Medicine Sciences, Jilin University, Changchun, China.; 5 Key Laboratory of Zoonosis Research, Ministry of Education, College of Veterinary Medicine, Jilin University.; 6 Ruminant Diseases Research Center, College of Life Sciences, Shandong Normal University, Jinan, China.

In our paper 1, the images of invaded BxPC-3 cells in Figure 2F, SW1990 cells in figure 5C and migrated SW1990 cells in figure 5B shared overlapped fields with images in other groups due to mis-paste. The Fig. 2 and Fig. 5 should be corrected as below. On account of either misused pictures didn't change the invasion or migrated abilities of indicated cells, we confirm the mistakes wouldn't affect the results and conclusions of our article. We apologize for any inconvenience or misunderstanding that the errors may have caused.

## Figures and Tables

**Figure 1:**
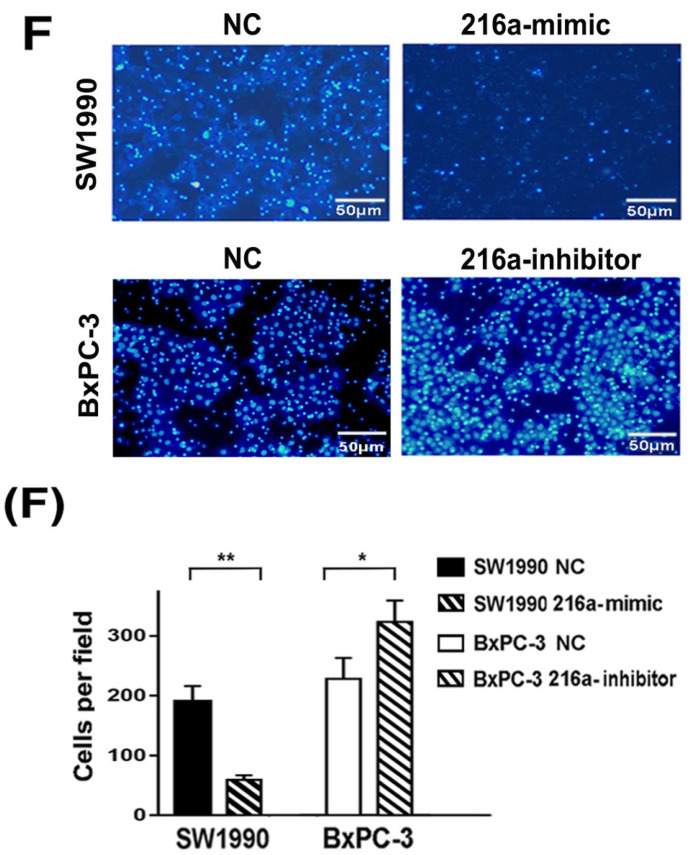
Corrected images for original Figure 2F.

**Figure 2:**
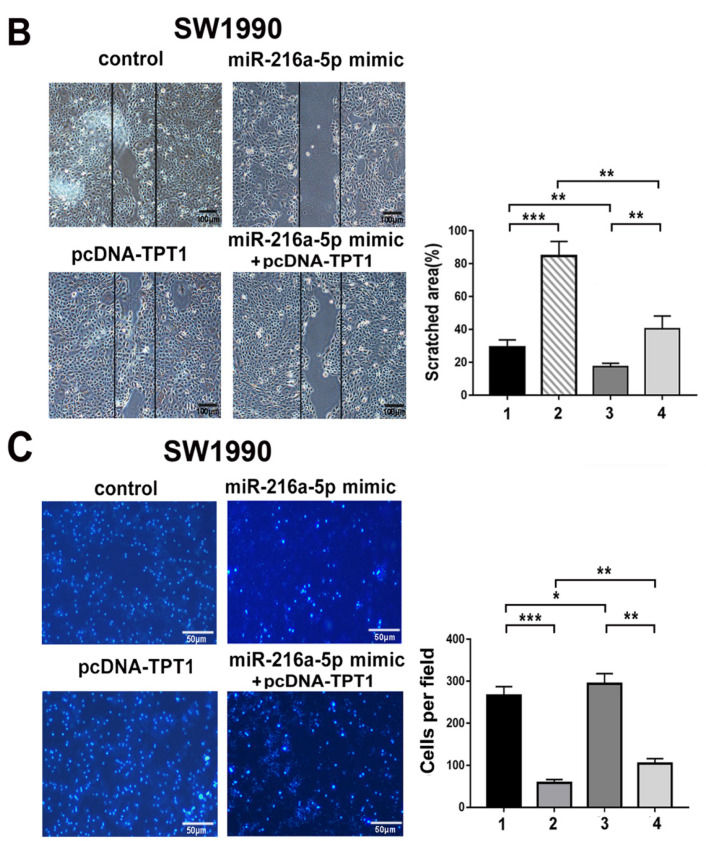
Corrected images for original Figure 5B and C.
