# Supplementary material for: MiR-216a-5p inhibits tumorigenesis in Pancreatic Cancer by targeting TPT1/mTORC1 and is mediated by LINC01133
Source: Int J Biol Sci. 2020 Jul 19;16(14):2612–27. doi: 10.7150/ijbs.46822 (PMC7415429; doi:10.7150/ijbs.46822)
Supplement: Supplementary file 1 — Supplementary figure S1. [file ijbsv16p2612s1.pdf]

Figure.S1

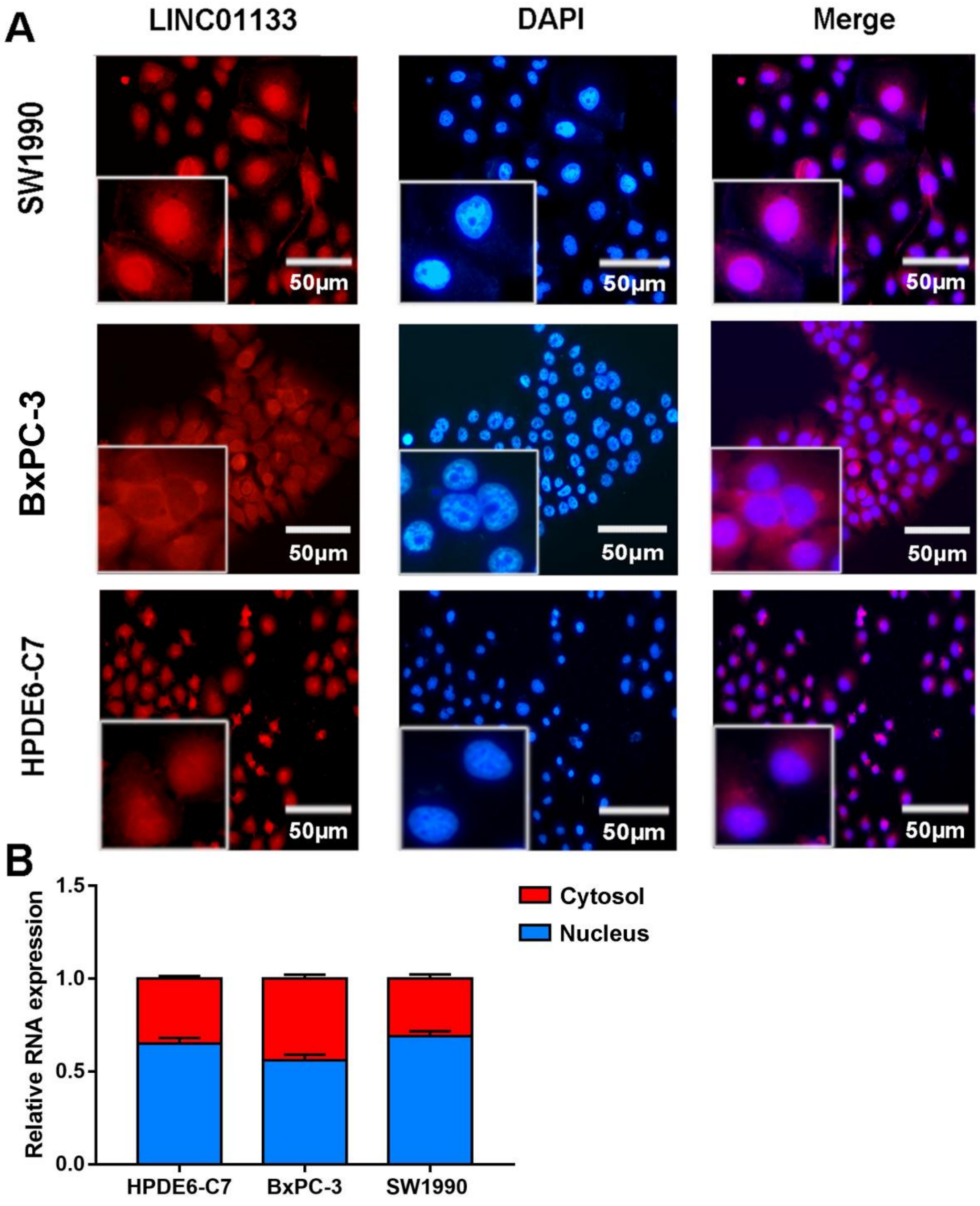

Figure S1. The distribution of LINC01133 in different pancreatic cell lines. (A) The distributions of LINC01133 in different cell lines were detected by FISH assays. Scale bar, 50μm. (B) The expression levels of LINC01133 in the subcelluar fractions of different cell lines were further confirmed by qRT-PCR.
